# Morpho-structural characteristics of feet in patients with rheumatoid arthritis: A cross sectional study

**DOI:** 10.7150/ijms.56935

**Published:** 2021-03-30

**Authors:** Andres Reinoso-Cobo, Pekka Anttila, Ana Belen Ortega-Avila, Pablo Cervera-Garvi, Eva Lopezosa-Reca, Ana Marchena-Rodriguez, Laura Ramos-Petersen, Gabriel Gijon-Nogueron

**Affiliations:** 1 Department of Nursing and Podiatry, Faculty of Health Sciences, University of Malaga, Arquitecto Francisco Peñalosa 3, Ampliación de Campus de Teatinos, 29071 Malaga, Spain.; 2 Applied Science of Metropolia Univesity, Podiatry Department, 01600 Helsinki, Finland.; 3 IBIMA. Malaga, Spain.; 4 Departamento of Podiatry. Faculty of Health Sciences. Universidad Católica San Antonio de Murcia. Campus de Los Jerónimos. Guadalupe 30107 Murcia Spain.

**Keywords:** rheumatoid arthritis, foot, Hallux Valgus, joint, foot posture

## Abstract

**Objective:** The aim of this study was to evaluate and classify the types and incidences of foot deformities in patients with Rheumatoid Arthritis (RA).

**Methods:** A cross-sectional study with convenience sample was obtained of 220 patients with foot pain and RA classification criteria (approved by the American College of Rheumatology and the European League against Rheumatism in 2010). A series of outcomes were assessed to measure the morphological characteristics of the feet. The Foot Posture Index (FPI), the Manchester Scale of Hallux Valgus and the Nijmegen classification of forefoot disorders were assessed.

**Results:** The most common foot posture according to the FPI assessment are the pronated position in the left foot (32.7% of participants) and the neutral position in the right foot (34.1% of participants). The disease progression causes more developed and serious foot deformities. 1.82% of patients present a severe level of Hallux Valgus before 10 years of disease evolution whereas 4.09% of patients present a severe level of Hallux Valgus after 10 years of disease evolution.

**Conclusions:** The most common foot type in patients with RA is the pronated foot type with deformities in the MTP joints without Hallux Valgus. However, a percentage of patients with RA presents supinated foot type. The evolution of the disease shows some morphological changes in terms of patient's feet. The presence of more developed foot deformities is increased, such us Hallux Valgus or MTP joints deformity (Grade 3 in the Nijmegen classification scale).

## Introduction

Rheumatoid arthritis (RA) is a chronic, progressive inflammatory disease that can cause limitations and difficulties in activities of daily living (ADLs) and pain. As a result, patients may present gait impairment and difficulties with self-care 1. The prevalence of RA in the population is approximately 7.7 per 1,000 and it is more prevalent in females, in whom two-thirds of new cases arise. The disease is prevalent in the fourth and fifth decades of life 2.

There are multiple affectations in the upper and the lower limb 3 as well as the quality of life of patients with RA is affected 4 and fatigue 5. RA is associated with significant pain and deformities, where individuals continue to perform activities with functional capacity restriction. Fatigue and functional disability ensue with the progression of the disease 6.

There is a high prevalence of foot involvement in RA with over 90% of patients reporting foot pain during the course of the disease 7. It has been suggested that erosive changes may occur in the joints of the hands and feet, particularly in the metatarsophalangeal (MTP) joints 8. Inflammation of foot joints and synovial tissues lead to articular damage and structural deformities. The most common foot deformities in patients with RA include dorsal subluxation of the lesser MTP joints, hallux valgus (HV), metatarsus primus varus (MPV), hallux rigidus, hindfoot valgus, pes planus (PP) and splaying of the forefoot (SF) 9.

In addition, there is involvement of the skin trophism with lesions such as callus formation and ulcers. Foot ulceration is estimated to affect 10-13% of patients with RA during the course of their disease. Furthermore, 47% of those affected patients experience multiple episodes of ulceration involving numerous sites on the foot 10. However, the deformities and morphological structure of the feet in the patients with RA remain unknown.

The aim of this study is to evaluate and classify the feet types and frequency of foot deformities in patients with RA.

## Materials and Methods

### Ethical approval

Institutional review board that approved the protocol for the study: Medical Research Ethics Committee of University of Malaga (CEUMA-91-2015-H) and PEIBA Andalucía (ARC0001), Spain.

### Design

A cross-sectional study.

### Participants

A convenience sample was obtained of 237 patients with foot pain and RA classification criteria (approved by the American College of Rheumatology and the European League Against Rheumatism in 2010) 11, of whom seventeen subsequently declined to participate, citing lack of time (the study questionnaire required 30 minutes to complete). The patients were enrolled at hospital outpatient clinics from January to December 2018. All the included participants in the study were adults (more than 18 years old) who had a history of subtalar and/or ankle and/or talonavicular or hindfoot pain, did not make daily use of walking aids, and were able to achieve the normal range of motions in the ankle, subtalar and midtarsal joints (even if maximum dorsiflexion, pronation or supination in these joints could not be produced, a sufficient range of motion was achieved by adjusting the dynamics, for example by reducing stride length) 12. The exclusion criteria applied were to present a concomitant musculoskeletal disease, central or peripheral nervous system disease and/or endocrine disorders (especially diabetes mellitus).

Patients who met the criteria for inclusion were approached by members of the rheumatology service at the Virgen de la Nieves Hospital (Granada, Spain), given an information sheet and invited to participate. Those participants who agreed were interviewed and given further details of the study. All participants provided written consent prior to starting the interviews.

### Data collection

#### Demographic and clinical characteristics

The demographic characteristics recorded included the patient's age, gender, disease duration and current therapy. The clinical data recorded to assess the patients disease status were the visual analogue scale for pain (VAS pain) 13, Disease Activity Score-28 (DAS28) 14 and Simplified Disease Activity Index (SDAI) 15.

A series of outcomes were assessed to measure the morphological characteristics of the feet. The Foot Posture Index (FPI) is a reliable instrument for this purpose 16. Furthermore, the Manchester Scale of Hallux Valgus 17 and the Nijmegen classification of forefoot disorders 9 were assessed.

### Procedure

Two researchers (ARC and GGN) independently interviewed the patients in order to obtain the study data. The clinical interview was conducted in one room, where the patients were asked to complete demographic characteristics. In a separate room, foot posture of each patient was measured. For that purpose, the FPI was assessed (intraclass correlation coefficient (ICC) for the clinician, 0.94-0.96). Each criterion was scored as -2, -1, 0, +1 or +2. The following FPI cut-off points, defining foot type category were used: a) highly supinated from -12 to -4, b) supinated from -3 to 0, c) neutral from 1 to 6, d) pronated from 6 to 10 and e) highly pronated from 11 to 12 (18). The presence/absence of hallux valgus was determined according to the Manchester Scale of Hallux Valgus (ICC for the instrument, 0.93-0.97). It is a clinical tool consisting of photographs of feet with four levels of hallux valgus: none, mild, moderate and severe (17). The Nijmegen classification of forefoot disorders is a classification system which can be used to grade the severity of the forefoot deformity. It presents four different levels to assess the deformity: Grade 0. No clinical changes in the metatarsophalangeal (MTP) joints, none or mild radiographic changes; Grade 1. Decreased mobility of one or more of the joints, particularly of plantarflexion, with the ability to reduce the plantar soft tissues under the metatarsal heads, and with adequate quality of the plantar soft tissues and/or radiographic erosive changes (Larsen 2-5) or evident intra-articular changes; Grade 2. Loss of plantar flexion in one or more of the MTP joints (up to 00), and loss of the ability to reduce the plantar soft tissues under the metatarsal heads, and/or with inadequate quality of the plantar soft tissues A. with a hallux valgus of more than 20º B. without a hallux valgus of more than 20º; Grade 3. Deep contracture in one or more MTP joint, with or without radiographic subluxation or dislocation A. with a hallux valgus of more than 20º. B. without a hallux valgus of more than 20º 9 (ICC for the clinician, 0.83-0.87).

### Statistical analysis

The results obtained are reported as the median and interquartile range, if the non-normal distribution of the variables, and as the mean and standard deviation (SD) due to the normal distribution. The normality of the distributions was examined by the Kolmogorov-Smirnov test and the intra-rater reliability of the measurement instruments was calculated by a two-way mixed-consistency ICC model. The bivariate analysis was performed with Student's t test and the non-parametric Wilcoxon test; for the association of qualitative variables, the chi-square test was used for the comparison of proportions. The significance level was set at p<0.05. All statistical analyses were conducted using SPSS v. 24.0 statistical software (SPSS Inc., Chicago, IL, USA).

## Results

In total, 220 patients with RA were analysed, (average of duration of RA in years, 15.44, SD 10.54 years), 173 patients were female. The values for median age and interquartile range (IR) were 59 and 16 years for the patients with RA. The median values for height and weight were 162 cm (IR: 10) and 65 kg (IR: 15). The patients with RA were treated with biological disease-modifying antirheumatic drugs (bDMARDs) (42%), methotrexate (35%) or nonsteroidal anti-inflammatory drugs (NSAIDs)/corticosteroids (20%). DAS 28 2.77 (SD 1.27) and SDAI 10.10 (SD 7.88).

**Figure 1:**
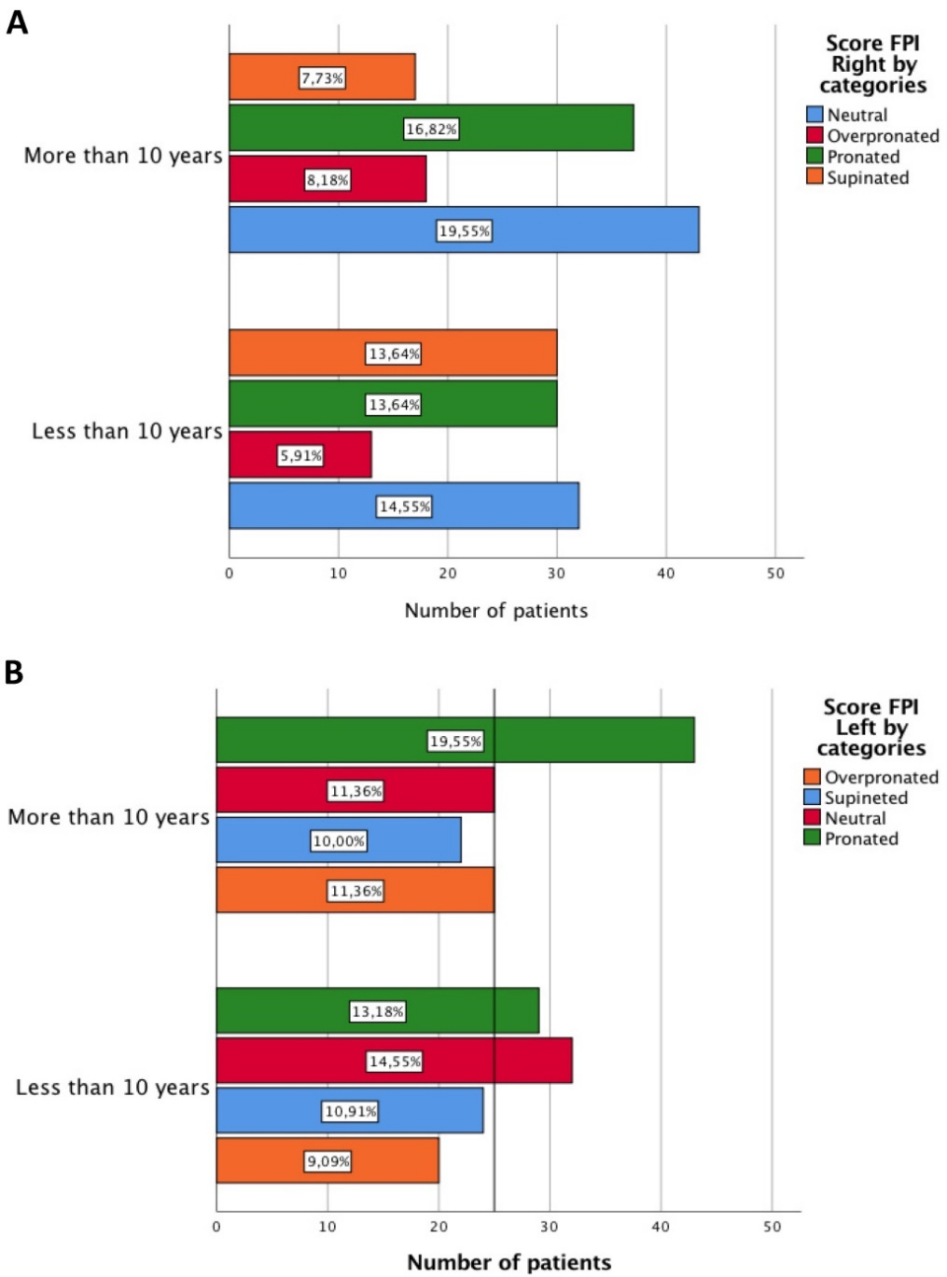
** A and B** Score of FPI differentiating according to years of disease evolution.

The most common foot posture according to the FPI assessment are the pronated position in the left foot (32.7% of participants) and the neutral position in the right foot (34.1% of participants) (Table 1). In patients with less than 10 years of RA in patients who were diagnosed less than 10 years ago, the right feet have shown a supinated position (13.64% of participants). On the other hand, in patients after 10 of RA evolution, the right feet having a neutral foot posture is most common (19.55% of participants). In the left feet different results are shown. In patients after 10 of RA evolution, the left feet have a pronated position (19.55% of participants) (Figure 1). Non-statistically significant differences were found using the chi-square test in boot feet (p=0.098 and p=0.257).

**Figure 2:**
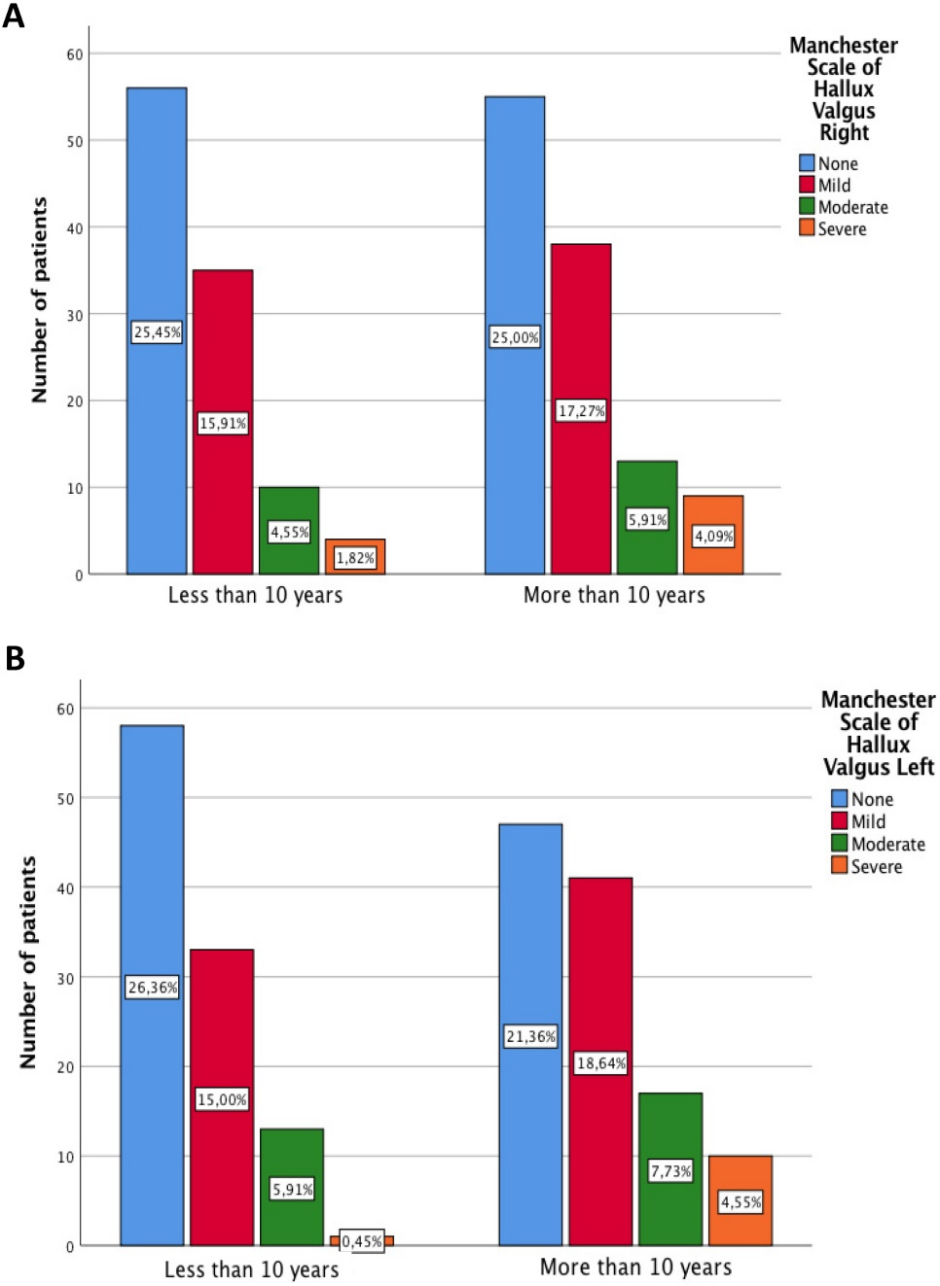
** A and B** Severity of Hallux Valgus by categories of Manchester Scale of HV differentiating according to years of disease evolution.

Regarding to the Hallux Valgus deformity, the progression of deformity overall is exacerbated by the hallux valgus deformity. 1.82% of patients present a severe level of Hallux Valgus before patients after 10 of RA evolution whereas 4.09% of patients present a severe level of Hallux Valgus after in patients after 10 of RA evolution. Statistically significant differences were not found using the chi-square test in the right foot (p=0.573), however, statistically significant differences were found in the left foot (p=0.024) (Figure 2A/B).

**Figure 3:**
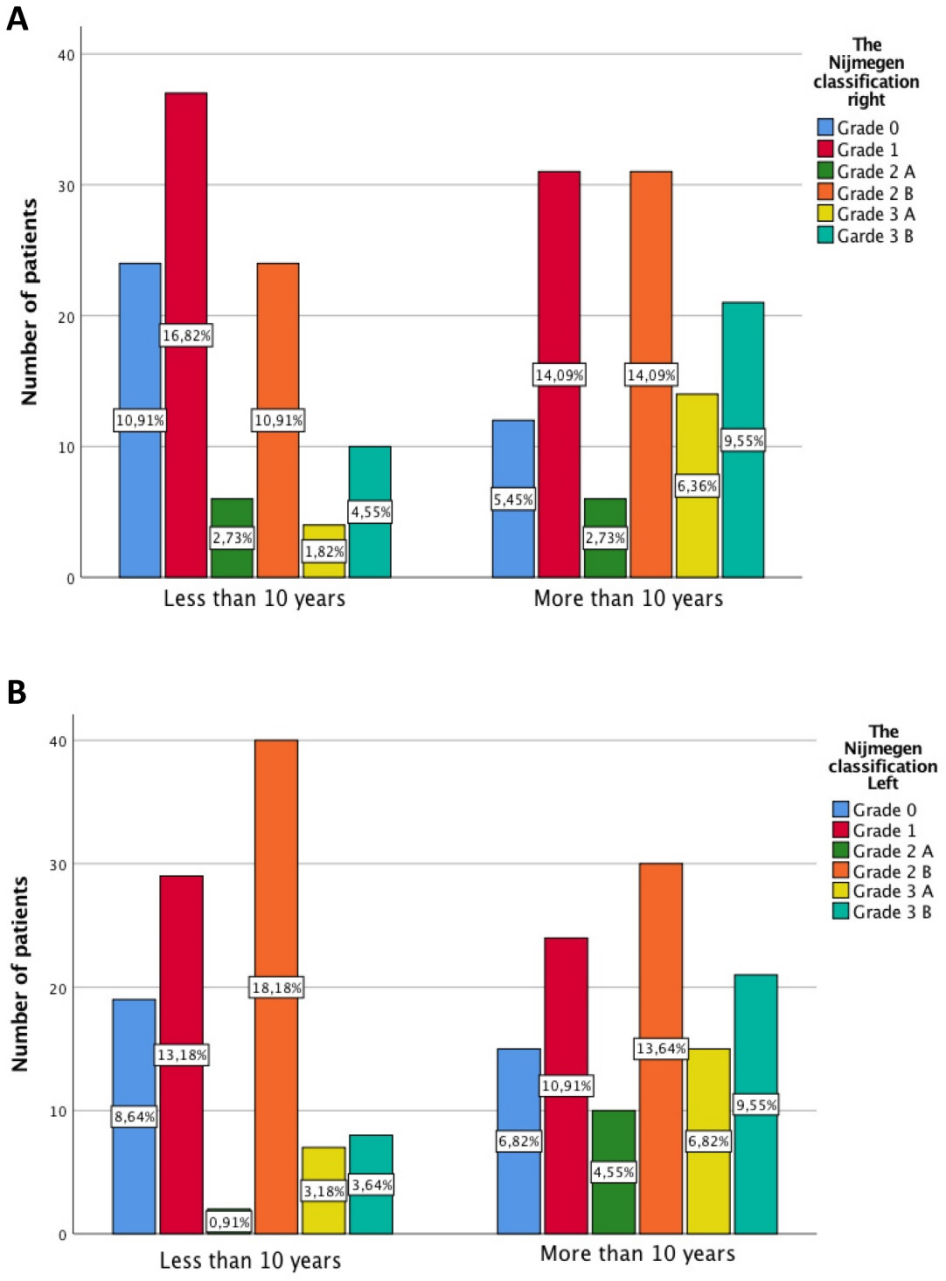
A and B Severity of metatarsophalangeal by categories of Nijmegen classification differentiating according to years of disease evolution.

In terms of lesser metatarsophalangeal deformities, statistically significant differences are presented in both feet (p=0.013 and p=0.007). The Grade 3 increases its percentage in both feet in patients with more than in patients after 10 of RA evolution (Figure 3A and B).

## Discussion

The aim of this cross-sectional study was to evaluate and classify the types and frequency of foot deformities in patients with RA. For the purpose of this study, the foot type defined by FPI, the Manchester Scale of Hallux Valgus 17 and the Nijmegen classification of forefoot disorders were assessed in patients with RA. As a result, the prevalence of HAV, forefoot deformities and foot type were described.

In previous studies, where different structural deformations in the foot were mentioned, the quantitative method used to demonstrate these structural changes were not described 19,20. In our study, to avoid the same bias, validated foot outcomes have been included, except the Nijmegen classification. Our results can be compared to the results of Biscontini at al. in 2009 21, where after using the FPI and the Manchester Hallux Valgus they concluded that patients with RA can frequently present with hallux valgus and pronated foot.

Both studies agree that feet of patients with RA suffer from a valgus pathology and forefoot deformity. However, several studies have demonstrated that some musculoskeletal alterations appear in the upper limbs, such as muscle atrophy, broken tendons, decreased joint range of motion, join instability, stiffness, pain and biomechanical impairment. All those alterations are not associated with the osteoarticular deformation of the foot 22,23. In addition, our study provides process evolution data with the differentiation between patients that present RA before and after 10 years. As opposed to previous studies such as Lee S.W. et al., which analysed the incidence of the foot and ankle affectation in patients with a mean disease evolution of only 8 years, quantifying the number of affected joints with pain, inflammation and radiological alterations in the foot and ankle 19.

As it has been described in our study, a large amount of patients, specially female, with RA suffer from hallux valgus and lesser toe deformities such as decreased mobility of one or more of the MTP joints, a reduction of soft tissues under MTP joints and/or radiographic erosive changes. It has been discussed in previous studies that foot symptoms are almost ubiquitous among patients with RA and are frequently severe, despite the exceptional progress in RA treatments 2.

However, Yano et al. only analysed the incidence of patients who presented alterations in the foot at the moment of the diagnosis, not reporting the prevalence of foot deformations 20. The first foot manifestations are usually in the forefoot, and these foot deformities get worse over time 2. This agrees with our results that show patients with RA present a higher levels of severe Hallux Valgus in patients after 10 of RA evolution than before 10 years of evolution. The development of HAV in patients with RA occurs in in a shorter period of time than in the general population due to the following structural alterations of the foot: increased medial pressure on the forefoot; synovitis in the first MTP joint, which causes the joint capsule laxity and instability; joint erosion which helps deviation in the transverse plane of the first MTP joint and increased laxity of the Lisfranc ligament increasing the intermetatarsal angle 24,25.

The FPI results from the included participants showed that patients with RA present a wide variety of foot types, including neutral, pronated, overpronated and supinated foot posture. The most common foot posture were the neutral and pronated position, showing an intention of higher values in the FPI after 10 years of disease evolution, which means pronated foot posture. Those results agree with previous studies which concluded that the hindfoot is frequently found in a valgus position in patients with RA. The presence of an alteration in joint alignment, a reduction in mobility and a change in pressure distribution to the medial aspect of foot progress in a valgus deformation 24-26.

## Strengths and weaknesses of the study

The strengths of this study include; validated and reliable outcome measures and questionnaires used to assess foot types and frequency of foot deformities in patients with RA. All the participants presented a longer duration of the disease of more than 10 years, which was useful to stablish a classification before and after 10 years of evolution. Also, a protocol has been followed with each patient. The limitations associated with this study must be acknowledged when interpreting the results. First, all the participants were mainly women, which correlated with the information that RA is most commonly found in females in Europe. Secondly, the influence of biologics treatment should have been assessed to differentiate the effects of the treatments on soft tissues (ligaments and muscles). Biologics treatment may influence soft tissues depending on how long the treatment has been used. As a result, biologics treatment may influence the appearance of pronated foot type.

## Future research

In further studies, analysing more homogeneous sample sizes are needed, as a non-homogeneous sample size may influence the results. Furthermore, studies with outcomes that allow making a relationship between pain, loss of functionality and/or quality of life, and feet deformity are required.

## Conclusions

The most frequently found foot type in patients with RA is the pronated foot, with deformities in the MTP joints without Hallux Valgus. However, a percentage of patients with RA present with a supinated foot.

The evolution of the disease shows some morphological changes in terms of patient's feet. An evolution of more severe stages of foot deformities are presented, such us Hallux Valgus or Grade 3 of MTP joints in the Nijmegen classification scale.

## Figures and Tables

**Table 1:** Characteristics of the sample in relation with the morphological foot in patients with RA

N (220)		Frequency (number)	Percentage (%)
**Foot Posture Index**			
Left	Supinated	46	20.9
	Neutral	57	25.9
	Pronated	72	32.7
	Overpronated	45	20.5
Right	Supinated	47	21.4
	Neutral	75	34.1
	Pronated	67	30.5
	Overpronated	31	14.1
**The Nijmegen classification**			
Left	Grade 0	34	15.5
	Grade 1	53	24.1
	Grade 2A	12	5.5
	Garde 2B	70	31.8
	Grade 3 A	22	10
	Grade 3B	29	13.2
Right	Grade 0	36	16.4
	Grade 1	68	30,9
	Grade 2A	12	5.5
	Garde 2B	55	25
	Grade 3 A	18	8.2
	Grade 3B	31	14.1
**Manchester Scale of Hallux Valgus**			
Left	None	105	47.7
	Mild	74	33.6
	Moderate	30	13.6
	Severe	11	5
Right	None	111	50.5
	Mild	73	33.2
	Moderate	23	10.5
	Severe	13	5.9
